# Anticholinergic burden quantified by anticholinergic risk scales and adverse outcomes in older people: a systematic review

**DOI:** 10.1186/s12877-015-0029-9

**Published:** 2015-03-25

**Authors:** Mohammed Saji Salahudeen, Stephen B Duffull, Prasad S Nishtala

**Affiliations:** School of Pharmacy, University of Otago, PO Box 56, Dunedin, 9054 New Zealand

**Keywords:** Anticholinergic scales, Antimuscarinic, Adverse outcomes, Older people, Anticholinergic burden, Expert opinion, Rating scale

## Abstract

**Background:**

The cumulative effect of taking multiple medicines with anticholinergic properties termed as anticholinergic burden can adversely impact cognition, physical function and increase the risk of mortality. Expert opinion derived risk scales are routinely used in research and clinical practice to quantify anticholinergic burden. These scales rank the anticholinergic activity of medicines into four categories, ranging from no anticholinergic activity (= 0) to definite/high anticholinergic activity (= 3). The aim of this systematic review was to compare anticholinergic burden quantified by the anticholinergic risk scales and evaluate associations with adverse outcomes in older people.

**Methods:**

We conducted a literature search in Ovid MEDLINE, EMBASE and PsycINFO from 1984-2014 to identify expert opinion derived anticholinergic risk scales. In addition to this, a citation analysis was performed in Web of Science and Google Scholar to track prospective citing of references of selected articles for assessment of individual scales for adverse anticholinergic outcomes. The primary outcomes of interest were functional and cognitive outcomes associated with anticholinergic burden in older people. The critical appraisals of the included studies were performed by two independent reviewers and the data were extracted onto standardised forms.

**Results:**

The primary electronic literature search identified a total of 1250 records in the 3 different databases. On the basis of full-text analysis, we identified 7 expert-based anticholinergic rating scales that met the inclusion criteria. The rating of anticholinergic activity for medicines among these rating scales was inconsistent. For example, quetiapine was rated as having high anticholinergic activity in one scale (n = 1), moderate in another scale (n = 1) and low in two other scales (n = 2). Citation analysis of the individual scales showed that the Anticholinergic Cognitive Burden (ACB) scale was the most frequently validated expert based anticholinergic scale for adverse outcomes (N = 13).

**Conclusions:**

In conclusion, there is not one standardised tool for measuring anticholinergic burden. Cohort studies have shown that higher anticholinergic burden is associated with negative brain effects, poorer cognitive and functional outcomes.

**Electronic supplementary material:**

The online version of this article (doi:10.1186/s12877-015-0029-9) contains supplementary material, which is available to authorized users.

## Background

Medicines with anticholinergic properties are frequently prescribed in the older population for various medical conditions [1]. The cumulative effect of taking one or more medicines with anticholinergic properties is referred to as anticholinergic burden [2]. The majority of medicines commonly prescribed to older people are not routinely recognised as having anticholinergic activity and empirically physicians prescribe these medicines based on their anticipated therapeutic benefits overlooking the risk of cumulative anticholinergic burden [3].

A number of studies have reported on the adverse effects associated with higher anticholinergic burden. Studies have found that anticholinergic medicines may adversely affect cognitive and physical function [4-13] and anticholinergic burden is a strong predictor of cognitive and physical impairments in older people living in both community and residential care [4-7,12,14]. A retrospective study conducted in Finland found that the use of medicines with anticholinergic properties is a strong independent predictor of mortality in older people [15,16]. More recently, several studies in the older population have also reported an association between anticholinergic exposure and mortality with an increased risk of hospitalisations [1,6,17,18].

Expert rating scales are routinely used in research and clinical practice to quantify anticholinergic burden. Expert opinion derived rating scales generally rank the anticholinergic activity of drugs into four categories, ranging from no known anticholinergic activity (= 0) to definite/high anticholinergic activity (= 3) [3,5,9,19,20]. The aim of this systematic review was to compare anticholinergic burden quantified by the anticholinergic risk scales and evaluate associations with adverse outcomes in older people.

## Methods

### Data sources and search strategy

A literature search in Ovid MEDLINE, EMBASE and PsycINFO covering the period 1984 - September 2014 was completed to identify anticholinergic risk scales using the keywords; (anticholinergic*.mp), AND (cogniti#.mp). The search was then limited to English language AND humans AND ("all aged (65 and over)" OR "aged (80 and over)"). The MEDLINE search strategy is presented in Additional file 1.

Following the primary literature search to identify the relevant studies, we carried out a citation analysis of individual rating scales to identify potential studies validating the association between anticholinergic burden quantified by the anticholinergic risk scales and adverse outcomes. The citation analysis was performed with the aid of Web of Science and Google Scholar to track prospective citing of references of selected articles.

**Figure 1:**
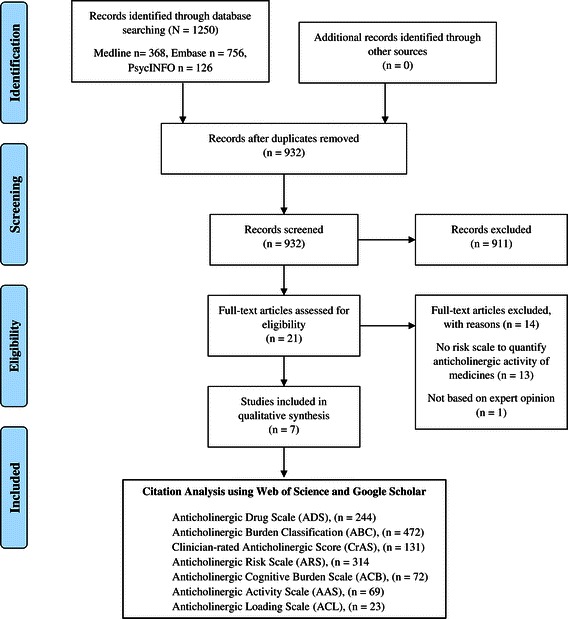
**PRISMA flow diagram of study selection process and citation analysis.**

Potentially relevant articles were selected according to the pre-defined inclusion and exclusion criteria. A flowchart of search strategy and citation search is depicted in Figure 1.

### Study screening and selection

Selecting the title and abstract of the publication, studies retrieved were screened by two independent reviewers for its eligibility for inclusion in the review process (M.S.S. and P.S.N.). The eligible studies were subject to a thorough full text analysis for relevance and pre-defined inclusion criteria. Studies that met the following criteria were included in the final review.

- The quantification tool was based on expert opinion.
- Studies that reported the use of expert opinion quantification scale/tool to measure anticholinergic burden.
- Studies that include participants of either sex, of mean age 65 years or older and living in primary care or nursing homes or hospitals.

We excluded articles in languages other than English, as well as case reports, commentaries, letters and editorials from the primary search and citation analysis. Anticholinergic rating scales based predominantly on serum anticholinergic activity (SAA) were also excluded from the review.

The primary aim of this review was to compare anticholinergic burden quantified by the anticholinergic risk scales and evaluate associations with physical, cognitive outcomes in older people.

Ethical approval was noted for all published papers included in the review.

### Data extraction and synthesis

Two reviewers (M.S.S. and P.S.N.) extracted data onto standardised format based on study population, study design, use of appropriate rating scales to quantify anticholinergic burden and outcome measures. The primary outcomes of interest were functional and cognitive outcomes associated with anticholinergic burden quantified by the expert opinion derived anticholinergic rating scales.

A citation analysis was performed to identify and compare the clinical utility of individual anticholinergic rating scales to quantify anticholinergic burden and to evaluate its association with adverse outcomes (cognitive, functional, mortality) in older people. Studies that used the rating scales for assessing the adverse outcomes in older people are reported in this review.

The quality of the included studies were critically appraised by two authors (M.S.S. and P.S.N.) using the United States Preventive Services Task Force (USPSTF) criteria [21]. The criteria to assess the internal validity of studies included: initial assembly and maintenance of comparable groups, measurements, clear definition of interventions, outcomes assessed and analysis. Critical appraisal scores derived from the USPSTF criteria were rated as poor, fair or good. Any differences between review authors concerning eligibility were reviewed by the third author (S.B.D.) and decisions were made by consensus.

## Results

The primary search using three databases identified a total of 7 scales as being relevant to this systematic review. A qualitative description of the included studies is shown in Table 1.

**Table 1:** **Overview of included anticholinergic rating scales**

Expert opinion based rating scales	Description	Number of anticholinergic activity medicines listed (N)
Carnahan USA, 2006 [9]	ADS is a four-point (0-3) scale that ranks anticholinergic drugs based on expert opinion	117
Ancelin France, 2006 [25]	ABC is a four-point scale (0-3) based on SAA and expert opinion	27
Han USA, 2008 [22]	CrAS is a four-point scale (0-3) based on pre-existing published anticholinergic scales and expert opinion	60
Rudolph USA, 2008 [19]	ARS is a four-point scale (0-3) based on extensive literature review and expert opinion	49
Boustani USA, 2008 [24]	ACB is a four-point (0-3) scale developed based on published data and expert opinion	88
Ehrt Norway, 2010 [26]	AAS is a five-point scale (0-4) based on existing evidence (Chew 2008 [38]) and expert opinion	99
Sittironnarit Australia, 2011 [23]	ACL is a four-point (0-3) scale based on pre-existing published anticholinergic scales and expert opinion	49

ADS = Anticholinergic Drug Scale; ABC = Anticholinergic Burden Classification; CrAS = Clinician-rated Anticholinergic Score; ARS = Anticholinergic Risk Scale; ACB = Anticholinergic Cognitive Burden Scale; AAS = Anticholinergic Activity Scale; ACL = Anticholinergic Loading Scale; SAA = Serum Anticholinergic Activity.

Points in rating scale represents, 0 = no anticholinergic activity, 1 = mild anticholinergic activity, 2 = moderate anticholinergic activity, and 3 = severe anticholinergic activity.

The primary electronic literature search identified a total of 1250 articles from 3 different databases such as Ovid MEDLINE, EMBASE, and PsycINFO. EndNote was used to eliminate duplicates and we considered 932 articles for screening. Out of 932 screened articles based on title and abstract, only 21 were eligible for full-text analysis. From the eligible 21 studies, 14 were excluded on full text analysis according to the set inclusion and exclusion criteria. Hence, in total, 7 studies were included in this review that considered expert opinion/s in the development of the anticholinergic rating scales [9,19,22-26]. Figure 1 depicts a flow-diagram of the identification, screening, eligibility and exclusion process.

The 7 scales ranked anticholinergic activity of medicines into four categories, ranging from no anticholinergic activity (= 0) to definite/high anticholinergic activity (= 3). The anticholinergic medicines described in the 7 rating scales were collated into a composite reference rating scale. The composite reference scale shows a total of 195 medicines derived from the 7 published scales that ranked anticholinergic activity from high to low as shown in Table 2.

**Table 2:** **A composite rating scale to categorise anticholinergic activity medicines (N = 195)**

High	Moderate	Low
Aceprometazine [25] (n = 1)		
Acepromazine [25] (n = 1)		
	*Alimemazine* (trimeprazine) [25] (n = 1)	*Alimemazine* [24] (n = 1)
*Alprazolam* [25] (n = 1)		*Alprazolam* [9,22-24] (n = 4)
	*Alverine* [25] (n = 1)	*Alverine* [24] (n = 1)
	*Amantadine* [19,24] (n = 2)	*Amantadine* [9,22] (n = 2)
Amitriptyline [9,19,22-26] (n = 7)		
Amoxapine [24,25] (n = 2)		
		Ampicillin [9] (n = 1)
		Aripiprazole [24] (n = 1)
		Asenapine [24] (n = 1)
		Atenolol [22,24] (n = 2)
Atropine [9,19,22-24] (n = 5)		
		Azathioprine [9] (n = 1)
	Baclofen [19,22] (n = 2)	
*Belladonna* [22,25] (n = 2)	*Belladonna* [24] (n = 1)	
		Benazepril [22] (n = 1)
Benzatropine/benztropine [9,19,24,26] (n = 4)		
		Betaxolol [22] (n = 1)
		Bisacodyl [23] (n = 1)
		Bromocriptine [9] (n = 1)
Brompheniramine [9,24] (n = 2)		
		Bupropion [22,24] (n = 2)
		Captopril [9,24] (n = 2)
	*Carbamazepine* [9,24] (n = 2)	*Carbamazepine* [22] (n = 1)
		carbidopa [19,22,23] (n = 3)
Carbinoxamine [9,24] (n = 2)		
Carisoprodol [19] (n = 1)		
		Cefamandole [9] (n = 1)
		Cefoxitin [9] (n = 1)
		Celecoxib [23] (n = 1)
		Cephalothin [9] (n = 1)
	*Cetirizine* [19,22,23] (n = 3)	*Cetirizine* [24] (n = 1)
		Chlordiazepoxide [9,22] (n = 2)
Chlorphenamine/chlorpheniramine [9,19,22-25] (n = 6)		
Chlorpromazine [9,19,22,24] (n = 4)		
		Chlorthalidone/chlortalidone [9,24] (n = 2)
	*Cimetidine* [9,19] (n = 2)	*Cimetidine* [24] (n = 1)
		Citalopram [23,26] (n = 2)
Clemastine [9,24] (n = 2)		
		Clidinium [24] (n = 1)
		Clindamycin [9] (n = 1)
Clomipramine [9,24,25] (n = 3)		
		Clonazepam [9,23] (n = 2)
*Clorazepate* [25] (n = 1)		*Clorazepate* [9,24] (n = 2)
*Clozapine* [9,24,26] (n = 3)	*Clozapine* [19] (n = 1)	
	*Codeine* [25] (n = 1)	*Codeine* [9,22-24] (n = 4)
*Colchicine* [25] (n = 1)		*Colchicine* [24] (n = 1)
		Cortisone [9] (n = 1)
	*Cyclobenzaprine* [9,19,24] (n = 3)	*Cyclobenzaprine* [22] (n = 1)
		Cycloserine [9] (n = 1)
		Cyclosporine [9] (n = 1)
*Cyproheptadine* [19,23] (n = 2)	*Cyproheptadine* [9,24] (n = 2)	
Darifenacin [9,24] (n = 2)		
*Desipramine* [9,24] (n = 2)	*Desipramine* [19,22] (n = 2)	
		Desloratadine [24] (n = 1)
		Dexamethasone [9] (n = 1)
Dexchlorpheniramine [23,25] (n = 2)		
		Dextromethorphan [22] (n = 1)
		Diazepam [9,22-24,26] (n = 5)
Dicyclomine [9,19,24] (n = 3)		
		Digitoxin [9] (n = 1)
*Digoxin* [25] (n = 1)		*Digoxin* [9,23,24,26] (n = 4)
		Diltiazem [9] (n = 1)
Dimenhydrinate [9,24] (n = 2)		
Diphenhydramine [9,19,22,24] (n = 4)		
		Dipyridamole [9,24] (n = 2)
	*Disopyramide* [9] (n = 1)	*Disopyramide* [24] (n = 1)
		Divalproex sodium [9] (n = 1)
		Domperidone [23] (n = 1)
	Dothiepin/dosulepin [23] (n = 1)	
Doxepin [9,22-24,26] (n = 5)		
Doxylamine [24] (n = 1)		
Emepronium [26] (n = 1)		
		Entacapone [19] (n = 1)
		Escitalopram [23] (n = 1)
		Estazolam [9] (n = 1)
		Famotidine [9] (n = 1)
		Fentanyl [9,24] (n = 2)
Fesoterodine [24] (n = 1)		
	Fexofenadine [22,23] (n = 2)	
Flavoxate [9,24] (n = 2)		
		Fluoxetine [9,22,23,26] (n = 4)
*Fluphenazine* [19,23] (n = 2)		*Fluphenazine* [9] (n = 1)
		Flurazepam [9] (n = 1)
		Fluticasone-salmeterol [9] (n = 1)
		Fluvoxamine [9,23,24,26] (n = 4)
*Furosemide* [25] (n = 1)		*Furosemide* [9,24] (n = 2)
		Gentamicin [9] (n = 1)
		Guaifenesin [22] (n = 1)
	*Haloperidol* [23] (n = 1)	*Haloperidol* [19,24] (n = 2)
Homatropine [22] (n = 1)		
		Hydralazine [9,24] (n = 2)
	Hydrocodone [22] (n = 1)	
		Hydrocortisone [9,24] (n = 2)
Hydroxyzine [9,19,24,25] (n = 4)		
Hyoscyamine [9,19,24] (n = 3)		
		Iloperidone [24] (n = 1)
Imipramine [9,19,22-25] (n = 6)		
Ipratropium [26] (n = 1)		
		Isosorbide [9,24] (n = 2)
		Ketotifen [9] (n = 1)
		Ketorolac [22] (n = 1)
		Ketotifen [9] (n = 1)
		Levocetirizine [24] (n = 1)
Levomepromazine [9,24,25] (n = 3)		
		Lithium [23] (n = 1)
	*Loperamide* [19] (n = 1)	*Loperamide* [9,22-24] (n = 4)
	*Loratadine* [19] (n = 1)	*Loratadine* [22-24] (n = 3)
		Lorazepam [9] (n = 1)
	Loxapine [9,24] (n = 2)	
		Lumiracoxib [23] (n = 1)
Maprotiline [25] (n = 1)		
Meclizine/meclizine [9,19,24] (n = 3)		
	Meperidine [9,24] (n = 2)	
		Metformin [23] (n = 1)
	Methadone [22] (n = 1)	
		Methocarbamol [19,22] (n = 2)
		Methotrexate [23] (n = 1)
	Methotrimeprazine [9,24] (n = 2)	
		Methylprednisolone [9] (n = 1)
		Metoclopramide [19,23] (n = 2)
		Metoprolol [22,24] (n = 2)
		Midazolam [9] (n = 1)
		Mirtazapine [19] (n = 1)
	Molindone [9,24] (n = 2)	
		Morphine [9,22,24] (n = 3)
		Naratriptan [23] (n = 1)
		Nefazodone [22] (n = 1)
	Nefopam [24] (n = 1)	
		Nifedipine [9,24] (n = 2)
		Nizatidine [9] (n = 1)
*Nortriptyline* [9,22,24] (n = 3)	*Nortriptyline* [19,26] (n = 2)	
*Olanzapine* [24] (n = 1)	*Olanzapine* [19,26] (n = 2)	*Olanzapine* [9,22] (n = 2)
Opipramol [25] (n = 1)		
Orphenadrine [9,24-26] (n = 4)		
		Oxazepam [9,23] (n = 2)
	Oxcarbazepine [9,24] (n = 2)	
*Oxybutynin* [9,19,24-26] (n = 5)	*Oxybutynin* [23] (n = 1)	
		Oxycodone [9,22,23] (n = 3)
		Paliperidone [24] (n = 1)
		Pancuronium [9] (n = 1)
*Paroxetine* [24] (n = 1)	*Paroxetine* [22,23,26] (n = 3)	*Paroxetine* [9,19] (n = 2)
*Perphenazine* [19,24] (n = 2)	*Perphenazine* [22] (n = 1)	*Perphenazine* [9] (n = 1)
		Phenelzine [9] (n = 1)
		Phenobarbital [22] (n = 1)
	Pimozide [9,24] (n = 2)	
		Piperacillin [9] (n = 1)
		Pramipexole [19] (n = 1)
		Prednisolone [9] (n = 1)
		Prednisone [9,24] (n = 2)
	*Prochlorperazine* [19,22,23] (n = 3)	*Prochlorperazine* [9] (n = 1)
Procyclidine [9] (n = 1)		
	Promazine [26] (n = 1)	
Promethazine [9,19,24] (n = 3)		
*Propantheline* [9,24] (n = 2)	*Propantheline* [22] (n = 1)	
Propiverine [24] (n = 1)		
	Propoxyphene [22] (n= 1)	
Protriptyline [9,23] (n = 2)		
	Pseudoephedrine [19,23] (n = 2)	
Pyrilamine [9] (n = 1)		
*Quetiapine* [24] (n = 1)	*Quetiapine* [22] (n = 1)	*Quetiapine* [19,26] (n = 2)
		Quinidine [24] (n = 1)
	*Ranitidine* [9,22] (n = 2)	*Ranitidine* [19,23,24,26] (n = 4)
Reglan [22] (n = 1)		
		Risperidone [19,22-24] (n = 4)
		Robitussin [22] (n = 1)
Scopolamine(hyoscine) [9,22,24] (n = 3)		
		Selegiline [19] (n = 1)
		Sertraline [9,22] (n = 2)
Solifenacin [24] (n = 1)		
		Sumatriptan [23] (n = 1)
		Temazepam [9,19,23] (n = 3)
	*Theophylline* [23,25] (n = 2)	*Theophylline* [9,24,26] (n = 3)
Thioridazine [9,19,22,24,26] (n = 5)		
*Thiothixene* [19] (n = 1)		*Thiothixene* [9] (n = 1)
Tizanidine [19] (n = 1)		
*Tolterodine* [9,22-25] (n = 5)	*Tolterodine* [19] (n = 1)	
	*Tramadol* [22,23] (n = 2)	*Tramadol* [9] (n = 1)
		Trandolapril [22] (n = 1)
		Trazodone [19,22,24] (n = 3)
		Triamcinolone [9] (n = 1)
		Triamterene [9,24] (n = 2)
		Triazolam [9,22] (n = 2)
*Trifluoperazine* [19,24] (n = 2)		*Trifluoperazine* [9] (n = 1)
Trihexyphenidyl [9,22,24-26] (n = 5)		
Trimipramine [9,24-26] (n = 4)		
Tropatepine [25] (n = 1)		
Trospium [24] (n = 1)		
		Valproic acid [9] (n = 1)
		Vancomycin [9] (n = 1)
		Venlafaxine [22-24] (n = 3)
		Warfarin [9] (n = 1)
		Ziprasidone [19] (n = 1)
		Zolmitriptan [23] (n = 1)

Medicines in *italics* denote inconsistent validation.

The Anticholinergic Drug Scale (ADS) developed by Carnahan et al. [9] based on expert consensus ranks medicines with anticholinergic properties in an ordinal fashion from 0 to 3, with 0 indicating no known anticholinergic activity and 3 indicating definite/high anticholinergic activity. This scale was initially referred to as the Clinician-rated Anticholinergic Scale (CrAS) modified version. An expert panel of geriatric psychiatrists identified and reviewed 340 medicines with known anticholinergic activity and assigned a score from 0 to 3 according to their clinical experience and the pharmacologic mechanism of each medicine. The ADS scale contains 117 medicines with known anticholinergic activity. The ADS scale has shown to be of utility in various clinical settings such as community, nursing homes, outpatient clinics, and hospitals. The adverse outcomes studied in these settings were mainly, cognitive, functional, risk of hospitalisation, and mortality.

The Anticholinergic Risk Scale (ARS) score was developed based on a ranking system developed by Rudolph et al. [19]. A literature review of 500 medicines known to possess anticholinergic activity was conducted by a group of geriatricians and pharmacists within the Veterans Affairs Boston Healthcare System. The authors considered the affinity for the muscarinic receptor, experimental reporting of anticholinergic activity, and literature review on anticholinergic adverse effects. This information was used to rank medicines for anticholinergic activity on a scale of 0 to 3, with 0 indicating no known anticholinergic activity and 3 indicating definite/high anticholinergic activity. A total of 49 medicines with known anticholinergic activity were reported in the ARS scale. The clinical outcomes validated using the ARS scale were cognitive, functional, quality of life, length of hospital stay, and mortality. The ARS was validated in a veteran’s population derived from a single medical centre limiting its external validity. Higher ARS scores in veteran and primary care patients were shown to be associated with anticholinergic adverse events [19].

Anticholinergic Cognitive Burden Scale (ACB) developed by Boustani et al. [24] is based on a systematic literature review of medicines with known anticholinergic activity. The ACB scale included medicines that were likely to have a negative impact on cognition [27,28]. A multi-disciplinary panel assessed individual drugs to have none, possible, or definite anticholinergic properties with a score ranging from 0 to 3. ACB scale reported 88 medicines with known anticholinergic activity. Studies that employed the ACB scale have shown that higher anticholinergic burden predicts cognitive impairment in older people. In addition, the study conducted by Pasina L et al. showed that anticholinergic burden quantified by the ACB scale predicted impairment in physical functioning [27].

Using similar methodologies other anticholinergic risk scales have been developed in Australia [23], Norway [26], France [25] and U.S.A. [22]. The CrAS scale by Han et al. was validated in palliative care and veteran home settings for cognitive and functional outcomes. The Anticholinergic Activity Scale (AAS) by Ehrt et al., and Anticholinergic Loading Scale (ACL) by Sittironnarit et al. were validated for only cognitive outcomes.

Citation analysis of individual anticholinergic rating scales show anticholinergic burden scores associated with adverse outcomes in older people in various clinical settings. An overview of the studies is presented in Table 3. The 38 studies retrieved comprised of 2 RCTs, 12 cross-sectional studies and 24 cohort studies that validated the 6 anticholinergic rating scales.

**Table 3:** **Summary of study characteristics and validation of anticholinergic rating scales and its association with adverse outcomes in older people**

Rating scales	Validation					
	Study design	Study population/setting	Study duration	Adverse outcome(s) studied	Significant association	Critical appraisal
**Carnahan USA, 2006 (ADS)**	Cross-sectional [9]	Long-term care residents (mean age 86), N = 279	1 month	SAA	+	Good
	RCT [39]	Nursing home residents (mean age 85), N = 64	11 months	Cognitive function	–	Good
	Cross-sectional [40]	Nursing home residents (mean age 73), N = 87	1 year	Cognitive function (MMSE)	–	Good
				Functional outcome (ADL)	–	
	Cross-sectional [41]	Community-dwelling (aged ≥75), N = 621	3 years	Adverse events	+	Fair
				Cognitive function (MMSE, GDP)	+	
				Functional outcome (ADL, IADL)	+	
	Longitudinal cohort [42]	Outpatient clinics (mean age 71.9 ± 7.3), N = 102	1 year	Cognitive function	+	Fair
	Prospective cohort [43]	Hospital inpatients with hip fracture (aged ≥65), N = 364	48 hours to 5 days	Cognitive function (delirium)	–	Fair
	Cross-sectional [44]	Hospital inpatients (mean age 67.9 ± 10.5), N = 450	28-30 days	Cognitive function	–	Fair
	Cross-sectional [45]	Hospitalised (mean age 84 ± 6), N = 71	1 year	Mortality	–	Fair
	Retrospective cohort [46]	Australian veterans (median age 80), N = 36015	2 years	Risk of hospitalisation for confusion or dementia	+	Good
**Han USA, 2008 (CrAS)**	Prospective cohort [22]	Community-dwelling men (aged ≥65), N = 544	2 years	Cognitive function (Verbal recall test)	+	Good
				Functional outcome (ADL)	+	
	RCT [47]	Palliative care (mean aged 71), N = 461	Mean survival was 8.9 weeks	Quality of life (McGill’s Quality of life index)	+	Fair
				Functional outcome (Karnofsky performance scale)	+	
	Prospective cohort [48]	Veteran home demented residents (mean age 83.4), N = 53	12 weeks	Cognitive function (MMSE)	–	Fair
				Functional outcome (BI)	–	
**Rudolph, USA 2008 (ARS)**	Retrospective and prospective cohort (one each) [19]	Hospital and long-term care facilities (aged ≥65), N = 132 and N = 117	9 months	Central adverse effects (Confusion, dizziness, falls)	+	Good
			10 months			
	Prospective cohort [15]	Hospital and long-term care (mean age 81.3), N = 1004	1 year	Mortality	–	Good
	Prospective cohort [29]	Hospitalised patients (mean age 83.6 ± 6.6), N = 362	5 months	Physical function (BI)	–	Good
				Mortality	–	
				LOS	–	
	Cohort study [49]	Hospitalised patients (mean age 83.6 ± 6.6), N = 362	5 months	Institutionalisation and comorbidities	+	Fair
	Cohort study [50]	Hospital rehabilitation unit (mean age 79 ± 7), N = 117	9 months	Functional outcome (BI)	+	Fair
				LOS	–	
	Cross-sectional [41]	Community-dwelling (aged ≥75), N = 621	3 years	Adverse events	+	Fair
				Cognitive function (MMSE, GDP)	+	
				Functional outcome (ADL, IADL)	+	
	Cross-sectional prospective [27]	Hospital (aged ≥65), N = 1380	3 months	Cognitive function (SBT)	+	Good
				Physical function (BI)	+	
	Longitudinal cohort [42]	Outpatient clinics (mean age 71.9 ± 7.3), N = 102	1 year	Cognitive function	+	Fair
	Cross-sectional [45]	Hospitalised (mean age 84 ± 6), N = 71	1 year	Mortality	+	Good
	Retrospective cohort [51]	National Health Insurance Research Database (aged ≥65), N = 54,888	1 year and 6 months	Emergency visit	+	Poor
				Hospitalisation	+	
				Constipation	+	
				Delirium	+	
				Cardiac arrhythmia	+	
				Cognitive impairment	–	
	Retrospective cohort [46]	Australian veterans (median age 80), N = 36015	2 years	Risk of hospitalisation for confusion or dementia	+	Good
**Boustani, USA 2008 (ACB)**	Cross-sectional [52]	Nursing home patient with dementia (aged ≥66), N = 87	2 years and 2 months	Quality of life: Multiple engagement observations	–	Fair
	Longitudinal cohort [32]	Community-dwelling (aged ≥70), N = 1652	6 years	Cognitive function	+	Good
	Observational cohort [53]	Hospitalised patients with cognitive impairment, N = 147 (aged ≥65)	Duration as of hospital admission	Cognitive function (Delirium using CAM)	–	Fair
	Part of longitudinal cohort [54]	Nursing & residential homes, day hospital and inpatients with AD (mean age 81 ± 7.4), N = 224	1 year and 6 months	Cognitive function (MMSE and SIB)	–	Fair
	Longitudinal cohort [33]	Community-dwelling and institutionalised patients (aged ≥65), N = 1304	2 years	Cognitive function	+	Good
				Mortality	+	
	Retrospective cohort [34]	Primary-care clinics (aged ≥65), N = 3690	1 year	Cognitive function (MCI)	+	Fair
	Prospective study [55]	Community-dwelling women (aged ≥75), N = 1429	5 years	Functional outcome (IADL)	+	Good
				Cognitive function (MMSE)	–	
	Longitudinal cohort [56]	Community-dwelling women (aged ≥75), N = 1484	10 years	Cognitive function (MCI)	+	Good
				Dementia	+	
	Cross-sectional prospective [27]	Hospital (aged ≥65), N = 1380	3 months	Cognitive function (SBT)	+	Good
				Physical function (BI)	+	
	Cohort study [57]	Community-dwelling without dementia (aged ≥65), N = 896	10 years	Cognitive function	+	Fair
	Retrospective study [58]	Hospital patients (aged ≥90), N = 419	3 months	Mortality	–	Fair
				LOS	–	
	Longitudinal cohort [42]	Outpatient clinics (mean age 71.9 ± 7.3), N = 102	1 year	Cognitive function	+	Fair
	Cross-sectional [45]	Hospitalised (mean age 84 ± 6), N = 71	1 year	Mortality	–	Good
**Ehrt, Norway 2010 (AAS)**	Longitudinal cohort [26]	Community-based PD patients (mean age 74.7), N = 78	8 years	Cognitive function (MMSE)	+	Good
**Sittironnarit Australia, 2011 (ACL)**	Cross-sectional [23]	Subjects in 3 groups; healthy controls (N = 211), MCI (N = 768) and AD (N = 133) of mean age 70.0 ± 7.0, 75.7 ± 7.6, and 78.0 ± 8.6 years	1 year and 10 months	Psychomotor speed and executive function	+	Good

SAA = Serum Anticholinergic Activity; ACE = Addenbrooke's Cognitive Examination; TMT = Trail Making Test; MMSE = Mini-Mental State Examination; CAM = Confusion Assessment Method; DSST = Digit Symbol Substitution Test; ADL = Activity of Daily Living; AD = Alzheimer’s Disease; IADL = Instrumental Activities of Daily Living; RCT = Randomised Controlled Trial; SIB = Severe Impairment Battery; SBT = Short Blessed Test; BI = Barthel Index; MCI = Mild Cognitive Impairment; PD = Parkinson’s Disease; LOS = Length of Stay; GDP = Geriatric Depression Scale; ADS = Anticholinergic Drug Scale; CrAS = Clinician-rated Anticholinergic Score; ARS = Anticholinergic Risk Scale; ACB = Anticholinergic Cognitive Burden Scale; AAS = Anticholinergic Activity Scale; ACL = Anticholinergic Loading Scale.

## Discussion

To our knowledge, this is the first systematic review that compare anticholinergic burden quantified by the anticholinergic risk scales and evaluated associations with adverse outcomes in older people.

The citation analysis of individual scales revealed that ACB scale by Boustani et al. [24] was the most frequently validated expert based anticholinergic scale on adverse outcomes (N = 13) followed by ARS [19] (N = 11], ADS by Carnahan et al. [9] (N = 9), CrAS scale by Han et al. [22] (N = 3) and 2 other scales [23,26]. The review found only two RCTs that showed an association with higher anticholinergic burden and adverse outcomes. The RCT that used the CrAS scale to quantify anticholinergic burden showed a positive association with functional outcome and quality of life and the RCT using the ADS scale reported a negative association with cognitive functioning. The adverse outcomes reported in the cohort studies included mainly cognitive and physical outcomes. The cognitive outcomes reported included mild-cognitive impairment, confusion, dizziness, falls, delirium, psychomotor speed and executive function. The functional outcomes reported were pertaining to activity of daily living, instrumental activity of daily living, quality of life, physical function, hospitalisation, length of hospital stay, and mortality. A detailed summary of validated studies for individual anticholinergic scales with critical appraisal is illustrated in Table 3.

Numerous studies have found an association between use of anticholinergic medicines and adverse outcomes related to physical function, cognition and falls in older people [2,4,29-31]. Pasina et al. compared anticholinergic burden derived from both ACB and ARS scales and found strong associations with impairment in cognitive and functional outcomes [27]. A study conducted by Rudolph et al. validated higher ARS scores were associated with increased risk of both peripheral and central anticholinergic adverse effects in older people [19]. Furthermore, Campbell et al. and Fox et al. conducted studies using ACB scale and found that the use of definite anticholinergics increased the risk of cognitive impairment among older people [32,33]. Overall, research has shown that use of medicines with anticholinergic activity among older people is associated with physical and cognitive decline [34,35].

The variability in quantification of anticholinergic burden between the 7 anticholinergic scales was not surprising considering that the drugs listed and anticholinergic activity ratings assigned varied considerably in the 7 scales. Expert consensus was derived from an interdisciplinary team that consisted of geriatricians, pharmacists, psychiatrists, general physicians, nurses and researchers who research aging. The subjective rating of the anticholinergic activity relied heavily on the panels knowledge of adverse effects associated with anticholinergic drugs. The 7 scales calculated the anticholinergic burden by summing the scores of each anticholinergic medicine with the assumption that the anticholinergic activity is linear and additive. The inclusion and rating of medicines with anticholinergic activity were predominantly influenced by subjective decisions. The final score was based on median values of ratings by each panel member. As a result, there are large differences between the published lists: e.g., beta‐blockers atenolol or metoprolol were assessed as anticholinergic drugs only in the studies of Han et al. [22] and Boustani et al. [24] (rating score 1) compared to the other rating scales.

Discrepancies in rating of anticholinergic medicines were noted in the scales. For example, quetiapine was reported as having high anticholinergic activity [24] in one scale (n = 1), moderate [22] in another scale (n = 1) and low [19,26] in two other scales (n = 2). A compiled reference composite scale which displays all 195 anticholinergic medicines extracted from the 7 anticholinergic rating scales is shown in Table 2 [9,19,22-26]. Similarly, a recent review collated a list of 100 medicines with definite or possible anticholinergic effects based on previously published list of anticholinergic risk scales and in conjunction with Martindale as a reference text [36].

The current anticholinergic risk scales tend to simplify the complexity of pharmacological mechanisms, which is quite challenging in geriatric risk assessment in older populations due to increased biological variation. However, there is no standardised consensus on how to quantify the anticholinergic burden and it is difficult to compare the study results from different methods and studies that have used the same method because different cut-off values for anticholinergic burden have been reported [3]. The majority of scales have not considered the multiple actions of medicines on the muscarinic receptor subtypes, the possible synergistic or antagonistic effects of medicines, or possible development of tolerance for anticholinergic medicine effects over time. Moreover, anticholinergic adverse effects are dose-dependent and the relative anticholinergic activities of various medicines are unlikely to be proportional to a 0:1:2:3 ratio. Also, there was no consensus on the definition of an anticholinergic medicine, and both the number and ranking of the anticholinergic drugs listed vary considerably between the scales [3,37]. Some scales considered the impact of different routes of administration when ranking the anticholinergic activity of medicines, while others excluded topical, ophthalmic, otologic and inhaled preparations.

This systematic review was comprehensive in that the electronic search conducted in 3 different databases endeavoured to identify all potential studies that met our eligibility criteria. The review explicitly looked into the anticholinergic scales partly or fully developed based on expert opinion. In addition to this, citation analysis provides further details about validation of the included scales. The objectives were clearly stated and the search methodology including the citation analysis was robust. A systematic approach was used to synthesise and characterise the findings of this review.

## Conclusions

Medicines with anticholinergic activity are frequently prescribed in older people, and several rating scales have been developed to quantify anticholinergic burden. There is not one standardised rating scale for measuring anticholinergic burden. The reference composite scale developed would be a useful tool for clinicians to identify medicines with anticholinergic activity.

## Additional file

Additional file 1:
**MEDLINE search strategy.**

## Abbreviations

ADS: Anticholinergic drug scale
ABC: Anticholinergic burden classification
CrAS: Clinician-rated anticholinergic score
ARS: Anticholinergic risk scale
ACB: Anticholinergic cognitive burden scale
AAS: Anticholinergic activity scale
ACL: Anticholinergic loading scale
SAA: Serum anticholinergic activity
