# Supplementary material for: Anticholinergic burden quantified by anticholinergic risk scales and adverse outcomes in older people: a systematic review
Source: BMC Geriatr. 2015 Mar 25;15:31. doi: 10.1186/s12877-015-0029-9 (PMC4377853; doi:10.1186/s12877-015-0029-9)
Supplement: Additional file 1: — MEDLINE search strategy. [file 12877_2015_29_MOESM1_ESM.docx]

**Additional file 1. MEDLINE search strategy**

| **Source** | **Search strategy: keyword/MeSH** |
| --- | --- |
| MEDLINE (Ovid SP) 1984 to September 2014 | 1. anticholinergic*.mp. |
|  | 1. antimuscarinic.mp. or exp Muscarinic Antagonists/ |
|  | 1. Cholinergic Antagonists/ |
|  | 1. or/1-4 |
|  | 1. Cognition Disorders/ or Cognition/ or cogniti*.mp. |
|  | 1. physical#.mp. |
|  | 1. or/5-6 |
|  | 1. 4 and 7 |
|  | 1. limit 8 to (English language and humans and ("all aged (65 and over)" or "aged (80 and over)") and last 20 years) |
